# Characterization of DNA Methylation in Circulating Tumor Cells

**DOI:** 10.3390/genes6041053

**Published:** 2015-10-21

**Authors:** Constantin F. Pixberg, Wolfgang A. Schulz, Nikolas H. Stoecklein, Rui P. L. Neves

**Affiliations:** 1 Department of General, Visceral and Pediatric Surgery, University Hospital and Medical Faculty of the Heinrich-Heine University Düsseldorf, Moorenstr. 5, 40225 Düsseldorf, Germany; E-Mails: constantin.pixberg@uni-duesseldorf.de (C.F.P.); nikolas.stoecklein@med.uni-duesseldorf.de (N.H.S.); 2 Department of Urology, University Hospital and Medical Faculty of the Heinrich-Heine University Düsseldorf, Universitätsstr. 1, 40225 Düsseldorf, Germany; E-Mail: wolfgang.schulz@uni-duesseldorf.de

**Keywords:** circulating tumor cells, CTC, molecular analyses, epigenetics, DNA methylation

## Abstract

Epigenetics contributes to molecular mechanisms leading to tumor cell transformation and systemic progression of cancer. However, the dynamics of epigenetic remodeling during metastasis remains unexplored. In this context, circulating tumor cells (CTCs) might enable a direct insight into epigenetic mechanisms relevant for metastasis by providing direct access to systemic cancer. CTCs can be used as prognostic markers in cancer patients and are regarded as potential metastatic precursor cells. However, despite substantial technical progress, the detection and molecular characterization of CTCs remain challenging, in particular the analysis of DNA methylation. As recent studies have started to address the epigenetic state of CTCs, we discuss here the potential of such investigations to elucidate mechanisms of metastasis and to develop tumor biomarkers.

## 1. Introduction

Metastasis is a multi-step process involving tumor cell migration, intravasation, survival in the blood stream, extravasation at a secondary site, and colonization. Despite significant progress in understanding of this complex metastatic cascade, there is still a significant lack of knowledge on the underlying molecular mechanisms, which is obviously a major obstacle for the development of more effective therapies. The fact that tumor-related mortality is due to metastasis in 90% of the patients emphasizes its tremendous clinical importance [1].

Systemic disease is driven by cells that have successfully accomplished at least some steps of the metastatic cascade. Compared to their primary tumors, these cells have usually accumulated additional genetic and phenotypic alterations as a result of ongoing genetic instability and the different selective pressures experienced in the various micro-environments [2]. In this respect, tumor cells that have left the primary tumor or metastases and that can be found in the circulation of patients (circulating tumor cells, CTCs) are an important source of information about this process. In addition to their heuristic value for understanding fundamental aspects of tumor progression, these cells have now been proven to be clinically relevant, and their molecular characterization is acknowledged as an opportunity for real-time monitoring of the disease progression and of the molecular evolution of metastatic systemic disease [3,4].

Another class of relevant blood-based biomarkers are cell-free nucleic acids, in particular DNA (cell-free DNA, cfDNA), which are found at increased concentrations in cancer patients. Different genetic and epigenetic analyses have already demonstrated the clinical potential of cfDNA [5]. cfDNA is thought to originate from apoptotic and necrotic cancer cells from the primary tumor site, CTCs, and distant metastases [6]. As cfDNA represents a source of tumor genetic material, its analysis can serve as a convenient alternative or a complement to CTCs-based assays. However, since the origin of cfDNA can be very heterogeneous and cannot be traced to specific tumor cell populations, analysis of CTCs is preferable especially when exploring mechanisms of metastasis. Despite its potential, cfDNA is outside the scope of our review focusing on CTCs; reviews on cfDNA can be found in [5,6].

So far, molecular analyses of CTCs have rather focused on their genomes, transcriptomes and proteomes. In contrast, the epigenetics of CTCs remains a largely unexplored field with great potential. Considering the accumulated evidence for the role of epigenetics, especially DNA methylation, in several cellular mechanisms of metastasis, the low number of studies addressing the epigenetics of CTCs is surprising. However, the few existing studies on DNA methylation of CTCs already provide interesting initial evidence for epigenetic remodeling in these cells and for the clinical potential of CTC-epigenetics. They will therefore certainly provide a basis for future investigations. In this review we discuss the potential of CTCs and DNA methylation analyses for elucidating the metastatic cascade. We then review in detail the existing literature on DNA methylation in CTCs, discuss the major technical challenges for their epigenetic analysis, and provide our perspective on the potential contained in a deeper epigenetic characterization of CTCs.

## 2. The Utility of CTCs as Tumor Biomarkers

Today, CTCs are investigated not only for their utility as biomarkers, but also because they provide an opportunity to directly access tumor material via a simple blood draw. The CellSearch^®^ system was the first semi-automated system designed specifically for the enrichment and enumeration of CTCs [7], and it is still the only system approved by the Federal Drug Agency (FDA) for that purpose. The clinical utility of CTCs detected by the CellSearch^®^ system is now widely recognized in different types of tumors including breast, prostate and colorectal carcinomas. In these cancers, CTC-counts above defined thresholds have prognostic impact, correlating with reduced progression-free and overall survival of the patients [8,9,10,11,12,13,14,15]. For breast cancer, several studies have moreover provided good evidence that CTCs might serve as markers for monitoring disease response to therapy at different disease stages and in diverse clinical settings. In the neoadjuvant setting, persistence of CTCs after therapy identifies a group of patients with significant increased risk for early metastatic relapse and reduced overall survival [16,17]. In the adjuvant setting, the large SUCCESS trial demonstrated that the detection of CTCs in 30 mL blood before therapy was significantly associated with poor disease-free survival, distant disease-free survival, breast cancer-specific survival, and overall survival [18]. In metastatic breast cancers, patients with five or more CTCs per 7.5 mL blood before initiation of therapy have worse progression-free survival and overall survival [8]. Also, in metastatic breast disease, CTC enumeration can help to monitor disease response to treatment [19,20]. In particular, the development or persistence of high CTC-counts during the course of therapy is a strong indicator of a worse outcome, shorter time to progression and overall survival [9,11,21]. Importantly, a multinational, randomized phase III trial involving 711 patients with metastatic castration-resistant prostate cancer (mCRPC), demonstrated the utility of CTC enumeration also as a clinical trial end point [22]. In particular, the study showed that patients with less than five CTCs in 7.5 mL blood at week 12 constituted a low-risk group with a significantly longer overall survival. In this study CTC counts combined with lactase dehydrogenase (LDL) levels (both measured at week 12 of treatment) satisfied all four Prentice criteria [23] as efficacy-response surrogate for overall survival, indicating that CTC enumeration at week 12 could be used as a valid end point criterion. Thus, CTC enumeration might allow obtaining information from clinical studies within much shorter time frames.

Despite the solidly documented prognostic value of CTCs, the predictive value of CTC enumeration *per se* could not yet been shown in prospective clinical trials [21]. In a manner, this inability underlines the relevance of going beyond enumeration to explore the molecular characteristics of CTCs. In fact, CTCs have been already investigated for the targets of molecular therapies. Notably, different studies revealed molecular differences in HER2 [24,25,26], ER and PR status between breast cancer CTCs and their matched primary tumors, and likewise in their gene expression profiles [27,28]. Strengthening the relevance of molecular characterization of CTCs as a complement to the analysis of primary tumors, Meng and colleagues verified that three out of four breast cancer patients with HER2-positive CTCs benefited from HER2-targeted therapy, even though the primary tumors were HER2-negative [24]. More recently, a prospective clinical study has demonstrated that the presence of androgen receptor splice variant 7 (AR-V7) mRNA in CTCs of patients with metastatic castration-resistant prostate cancer (mCRPC) was consistently associated with resistance to two compounds targeting the AR-response (enzalutamide and abiraterone) [29]. In this study involving 62 CTC-positive mCRPC patients, AR-V7 could be detected in baseline samples of 18 patients none of whom benefited from either therapy. In addition, positivity for AR-V7 in secondary end points was statistically significant associated with shorter overall survival and other outcome parameters. Further prospective studies involving larger cohorts are underway to validate the important clinical implications of this study.

Importantly, besides their clinical relevance, CTCs constitute also an important source of material to address the metastatic cascade. Recently, evidence was obtained that patient-derived CTCs comprise indeed tumor initiating cells by injecting them directly into immunocompromised mice [30]. In addition, among patient CTCs, a subset of EpCAM+/CD44+/CD47+/MET+ CTCs had the potential to seed metastases in bone, lung and liver [31]. Further, one other independent study has also demonstrated that EpCAM-negative cells expressing HER2, EGFR, HPSE, and NOTCH1, isolated from patient-derived CTC cell lines have the potential to form metastases in lung and brain of mice [32].

The potential of exploring CTCs for a better understanding of the metastatic cascade and for clinical applications is hampered by technical difficulties. Even in metastatic patients, CTCs are extremely rare (their frequency can be as low as one CTC per 10^5^–10^7^ mononuclear cells) [33], which imposes limitations for their detection and detailed characterization. One factor contributing to the low number of CTCs is the commonly used low blood volume of 7.5–10 mL. One approach to circumvent this limitation employs diagnostic leukapheresis (DLA), which allows screening of up to 2.5 L of peripheral blood for CTCs and substantially enhances the CTC detection frequency [34]. Other limitations are associated with the enrichment and detection of CTCs. To address these, several methodologies and devices have been developed that exploit phenotypical (e.g., expression of surface markers) and/or morphological (e.g., size, density, deformability) differences between CTCs and normal blood cells [35,36]. The CellSearch^®^ system used in the majority of clinical CTC studies relies on EpCAM-based immunomagnetic enrichment and detection of nucleated, cytokeratin 8/18/19-positive, but CD45-negative cells [7]. The specificity of this system was demonstrated by the finding that virtually all CTCs enriched by CellSearch^®^ display aCGH profiles with typical structural chromosomal changes expected for the respective tumors [37,38]. Many other systems also rely on EpCAM for the enrichment of CTCs [36], such as the Isoflux™ system based on microfluidics [39], the MagSweper [40], the CTC-iChip, which combines EpCAM-based enrichment and morphological criteria for the identification of CTCs [41], or the CellCollector^®^, a functionalized wire inserted into the cubital vein for 30 min for *in vivo* capturing of CTCs [42]. For a comprehensive review on methods available for CTC enrichment, please see Yap *et al.*, 2014 [36].

A critically discussed issue with all EpCAM-based capture strategies is that EpCAM on carcinoma cells, specifically those in the circulation, may become downregulated [43]. Evidence from metastasizing xenograft models suggests that this downregulation may happen rapidly, within the first hour of circulation in blood [44]. There are several explanations for lack of EpCAM expression on CTCs [45], including epithelial-to-mesenchymal transition (EMT), which is discussed as an important mechanism for dissemination and metastasis. In fact, subsets of CTCs with a mesenchymal-like phenotype can be observed in the circulation [46] and seem to accrue with advanced disease stages [47,48]. This phenotypical heterogeneity among CTCs imposes obvious limitations on any technique for their enrichment and identification [49,50]. To circumvent the dependency on EpCAM, other methods have been developed for less restrictive enrichment of CTCs [36,51]. These include filter systems that rely on the larger size of CTCs like the Rarecells^®^ device [52], the ScreenCell^®^ system [53], the CellSieve™ system [54], or systems that rely on functional properties of CTCs like the Vita-Assay™, which is based on the ability of CTC to adhere to and invade a matrix membrane [55]. Following enrichment, the distinction of CTCs from co-enriched normal blood cells again requires tumor-specific markers, which are typically identified by immunostaining or *in situ* hybridization. Like the biological markers used for enrichment, the markers used for the identification of CTCs are not undisputed and need more validation. To achieve standardization of technologies, markers and assays for CTC detection and analysis, more than 30 European academic and private institutions have joined efforts in an Innovative Medicines Initiative (IMI) called CANCER-ID (started in January 2015).

In conclusion, CTCs seem to be relevant for systemic progression and can provide access to the characteristics of the systemic disease in the sense of a “liquid biopsy” for diagnosis, prognosis and eventually guidance of therapeutic decisions. Furthermore, their direct molecular characterization may contribute to a better understanding of the metastatic process.

## 3. Biological and Clinical Relevance of DNA Methylation in Cancer

DNA methylation acts as a major epigenetic mechanism within the complex network of mechanisms regulating gene expression [56]. The chemically stable addition of a methyl group to cytosines interferes with the capacity of several proteins to bind DNA [57]. Methylation in CpG-rich gene promoters serves as a recognition signal for proteins mediating transcriptional repression and constitutes a mechanism for stable gene silencing, whereas methylation of intragenic regions usually correlates with increased gene transcription [58,59]. For the regulation of tissue-specific expression, DNA methylation levels at enhancer sequences appear to be crucial. Methylation in repetitive transposable elements plays an important role in their silencing and contributes to global genomic stability [60]. Physiologically, methylation patterns are established during early embryogenesis and contribute to lineage commitment and cellular differentiation. These patterns are faithfully maintained during DNA replication by specialized DNA (cytosine-5-)-methyltransferases (DNMTs), but under several pathological conditions global changes in the epigenetic landscape take place [61,62]. In tumours, global methylation levels are often diminished (global hypomethylation) whereas simultaneously methylation is gained at specific loci (local hypermethylation). In various cancers, hypomethylation is observed at normally methylated retroelements [60,63], while hundreds to thousands of individual genomic loci become hyper- or hypomethylated compared to matched normal tissues [64].

Interestingly, methylation patterns established during tumorigenic cellular transformation and subsequent tumor progression are not random, suggesting that the rearrangements of the methylation landscape during carcinogenesis are orchestrated in a tumor-specific manner [65]. Examples of this orchestrated DNA methylation remodeling can be found in several genes involved in EMT [66], a process thought to drive tumor cell dissemination [67], acquisition of stem cell properties [68], and to be crucial for the fate of CTCs [69]. These genes comprise among others those encoding E-cadherin (*CDH1*), TWIST, Vimentin (*VIM*), N-cadherin (*CDH2*) and the miR-200 family of miRNAs [70,71,72,73,74]. Notably, changes in DNA methylation during the induction of EMT in MDCK cells by TGFβ and its reversion (MET) are dynamic [66,75]. Stable alterations in DNA methylation can therefore serve as a biomarker for cancer, but some changes in DNA methylation might also be used to follow the dynamics of cellular phenotypic plasticity as it occurs during metastasis.

Another example of a group of epigenetically regulated genes relevant for metastasis are “metastasis suppressor genes”. These are defined as genes that do not substantially affect tumor growth at the primary site, but must be inactivated to permit metastasis [76]. Typically, inactivation of these genes occurs not by mutations, but by epigenetic mechanisms, which may be dynamic during tumor progression. For instance, mice lacking the *Epb41l3* gene do not develop cancers spontaneously, but metastasis of autochthonous prostate cancer is enhanced [77]. This gene is downregulated by hypermethylation in several human carcinomas, including prostate cancer [78]. It will be highly interesting to find out whether methylation of such genes is accentuated also in CTCs.

The importance of DNA methylation in cancer has led to the development and testing of several drugs that interfere with the regulation of this epigenetic modification. The general idea of epigenetic therapies is the re-establishment of more normal patterns of gene expression which had become deregulated in cancer cells. Azacitidine (Vidaza) and its deoxy derivative 5-aza-2'-deoxycytidine (Decitabine) (both FDA-cleared epigenetic drugs) are nucleoside analogues that are incorporated into DNA during replication instead of cytidine and, by inactivating and depleting DNMTs, lead to global hypomethylation of the genome. These drugs, alone and in combination with other chemotherapeutic agents, have proven their benefits especially in patients with hematologic tumors (e.g., myelodysplastic syndrome [79,80], and acute myeloid leukemia [81,82,83]). In solid tumors, their application is more controversial [84,85]. However, in the management of some refractory advanced solid tumours, low-dose Decitabine-based chemoimmunotherapy has shown promise [86].

The chemical stability of cytosine methylation, the biological stability of methylation patterns through cell division, and the tumor-specific re-organization of the methylation landscape, taken together, make DNA methylation an attractive source of tumor biomarkers. One good example is the promoter methylation of the DNA repair gene *MGMT* coding for O-6-methylguanine-DNA methyltransferase. Glioblastomas [87] and stage IV melanomas [88] with *MGMT* methylation respond better to treatment with alkylating agents (temozolomide and dacarbazine), and *MGMT* methylation is an independent variable associated with disease-free [88] and overall survival [87]. In fact, *MGMT* methylation is now routinely used for selection of glioblastoma patients for temozolomide treatment [89]. In colorectal cancer as well, *MGMT* hypermethylation is significantly associated with a favourable clinical response to dacarbazine [90]. Several other methylation events have also been identified as prognostic markers. For instance, methylation of the *VIM* gene encoding the EMT indicator vimentin is associated with a markedly decreased survival in breast cancer [72]. Contrarily, it is also suggested as a biomarker of favorable prognosis in pancreatic cancer [91] and uterine cervical squamous cell carcinoma [92]. Hypermethylation of *VIM* can be used for detection of colorectal cancer using stool samples [93] and of urothelial carcinoma using urine [94,95].

Interestingly, in most cancers, more genes are affected by alterations in DNA methylation than by somatic genetic mutations [96], offering a much wider range of targets to design assays. Moreover, in contrast to genetic mutations, DNA methylation can be reversed and tumor regression might also involve the reversal of such biomarks. The dynamics of epigenetic biomarkers might therefore provide a good tool for monitoring disease progression and response to treatment.

**Figure 1:**
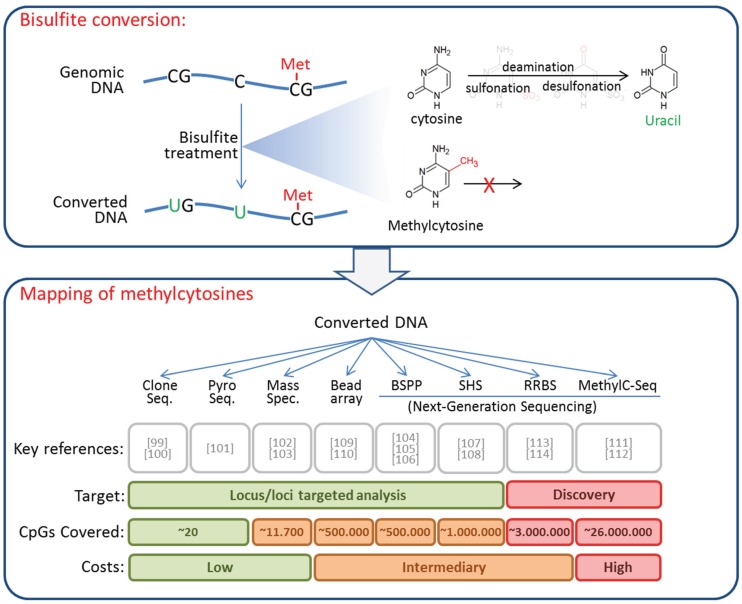
Bisulfite conversion of DNA and methods used for high resolution mapping of methylcytosines. (**Upper panel**) DNA conversion upon treatment with bisulfite. Bisulfite reacts with non-methylated cytosine residues converting them to uracils by initial sulfonation, hydrolytic deamination and final desulfonation. Methylcytosines essentially do not react with bisulfite. After conversion, uracil residues indicate non-methylated cytosines, while cytosines indicate methylcytosines in the original DNA sequence; (**Lower panel**) Converted DNA can be analyzed by multiple techniques discriminating between cytosines and uracils to detect or precisely map methylcytosines. These include clone sequencing (Clone Seq.), pyrosequencing (Pyro Seq.), MALDI-TOF mass spectroscopy (Mass Spec.), bead arrays, bisulfite padlock probes (BSPP), solution hybrid selection (SHS), reduced-representation bisulfite sequencing (RRBS), and whole genome sequencing (MethylC-Seq). These methods differ in the size of the target sequence, the number of CpGs covered, as well as in their cost.

Several robust technologies are now available for analyzing DNA methylation in tumor material and body fluids. The choice between them depends on the scope, resolution, available biological material, and involved labor and costs [97,98]. The methods range from locus-specific, through large-scale analysis, to whole methylome approaches. Some of the methods allow precise determination of the methylation status at individual CG-dinucleotides, while others provide only estimates of methylation levels across defined genomic regions or the entire genome. Most current techniques for high-resolution methylation analysis require treatment of DNA with sodium bisulfite, which converts unmethylated cytosine to uracil, whereas methylcytosine remains unaffected. Subsequent to conversion, different methods can be used to discriminate between uracil and cytosine nucleotides, and to infer the presence of unmethylated or methylated cytosines in the original sequence (Figure 1).

Standard Sanger sequencing after cloning [99,100] or the more quantitative pyro-sequencing technique [101] provide single CpG resolution across a few hundred bps for individual loci. A matrix-assisted laser desorption/ionization time of flight (MALDI-TOF) mass spectrometry-based technique has the advantage of easy automatization [102,103]. Several bisulfite sequencing techniques are available for intermediate scale analyses of multiple pre-selected CpGs. They rely on the capture of target regions by padlock probes [104,105,106] or solution hybrid selection [107,108], and can be coupled to next generation sequencing to achieve high throughput. Another widely used technique at intermediate scale is hybridization to methylation bead arrays covering approximately 500,000 CpG sites across the genome [109,110]. Comprehensive, but expensive whole-genome bisulfite sequencing [111,112] and the less costly reduced representation bisulfite sequencing (RRBS) [113,114] are commonly used for high resolution analysis of entire genomes (methylomes).

The application of established epigenetic methods to rare cell populations, such as CTCs, remains very challenging, especially at the single-cell level. For various genetic and genomic analyses, DNA from single cells can be reliably amplified. However, DNA methylation patterns are not maintained during DNA amplification, and analysis of methylation therefore requires bisulfite treatment of DNA prior to amplification. This poses a technical challenge, especially for samples composed of few cells as a minute amount of DNA needs to be carried through several treatment and purification steps before the actual analyses. In addition, bisulfite treatment causes random DNA fragmentation, which can be a limitation for subsequent analyses. Nevertheless, some bisulfite-based protocols have already been successfully applied to few or even single-cells by RRBS [115] or single-cell locus-specific bisulfite sequencing (SLBS) [116]. These studies have addressed DNA methylation changes during embryonic development illustrating the potential of these protocols for detecting inter- and intracellular methylation heterogeneity. Further development of these techniques will be needed for the systematic and robust analysis of DNA methylation which would be necessary to explore the epigenetic heterogeneity of tumor cells and CTCs at the single-cell level.

## 4. DNA Methylation in CTCs

The number of studies on methylation analysis of CTCs is so far small (Table 1). This is most likely explained by the combined technical challenges of CTC isolation and DNA methylation analyses on extremely rare cells.

In a series of three pioneering studies, Chimonidou and colleagues [117,118,119] investigated the methylation status of three tumor-associated genes in tumor cells from the blood of patients with breast cancer. The selected genes comprised Cystatin M6 (*CST6*), breast cancer metastasis suppressor 1 (*BRMS1*), and SRY (sex-determining region Y)-box 17 (*SOX17*). Cystatin M6 (E/M), an endogenous inhibitor of cathepsins B and L [120,121], is a postulated tumor suppressor in breast cancer [121,122]. Epigenetic silencing of *CST6* had already been associated with unfavorable prognosis in operable breast cancer [123,124]. BRMS1 was described as a metastases-suppressing protein [125] whose expression is modulated by DNA methylation [126]. Its reduced expression was correlated with poor prognosis [127,128] and predicted shorter disease-free survival in breast cancer [129]. *SOX17*, controlled by DNA methylation, encodes a transcription factor with a potential tumor suppressor function by antagonizing WNT-signaling [130,131]. Its reduced expression was associated with tumor progression and poor prognosis in breast cancer [132], and with patient survival in melanoma [133]. Considering the evidence pointing to the biological and clinical importance of epigenetic remodeling in these genes, Chimonidou and colleagues investigated whether methylation of these genes could also be detected in breast CTCs and could yield prognostic information. In a first study [117], the authors enriched EpCAM-expressing cells from the blood of patients with early and metastatic breast cancer or healthy controls by immunomagnetic capturing, and analyzed the methylation at the promoter regions of *CST6*, *BRMS1*, and *SOX17* by methylation-specific PCR (MSP) categorizing the samples as positive/negative. The frequency of methylation at the three gene promoters was significantly higher in EpCAM-enriched cells from patients than in those from healthy donors [117]. Notably, the authors observed that the frequency of methylation at these genes tended to increase from patients with operable tumors to patients with metastatic disease [117].

**Table 1:** Technical details and key findings of publications addressing DNA methylation in CTCs.

Reference	Entity	CTC Preparation		Methylation Analysis		Key Findings
		Enrichment	Detection and Isolation	Locus	Method	
Chimonidou, Strati *et al.*, 2011 [117]	BC (Human)	Density centrifugation + EpCAM-coated beads	-	CST6, BRMS1, SOX17	MSP	Positive correlation with presence and stage of disease.
Chimonidou, Strati *et al.*, 2013 [118]	BC (Human)	Density centrifugation + EpCAM-coated beads	-	SOX17	MSP (real-time)	Strong correlation between methylation detected in CTCs and cfDNA.
Chimonidou, Kallergi *et al.*, 2013 [119]	BC (Human)	Density centrifugation	Detection of CK+ cells on cytospins by ICC and scratching	BRMS1	MSP	Weak correlation between methylation and protein expression. Discordant methylation in CTCs and patient-matched PTs.
Friedlander, Ngo *et al.*, 2014 [134]	mCRPC (Human)	CAM	-	Genome wide (27000 CpG)	Methylation array	Correlation between methylation in CTCs and non-matched PTs. Hypermethylation in apoptosis, angiogenesis, and VEGF pathway genes.
Ogunwobi, Pussyk *et al.*, 2013 [135]	HCC (Murine cell line)	Implantation of tumor cells in mice, and establishment of CTC lines		HGF, c-Met	HRM and Pyrosequencing	HGF and c-MET overexpression in the CTC-lines correlated with hypomethylation of their promoters.

Notes: BC—breast cancer; mCRPC—metastatic castration-resistant prostate cancer; HCC—hepatocellular carcinoma; CAM—cell-adhesion matrix; MSP—methylation-specific PCR; HRM—high resolution melting; cfDNA—cell-free DNA; PTs—primary tumors.

Cell-free DNA (cfDNA) circulating in blood, too, has been proposed as a relevant source of tumor material and biomarkers [136], but its association with CTCs is still obscure. In a second study [118], Chimonidou and colleagues therefore explored the correlation between *SOX17* promoter methylation in EpCAM-expressing cells enriched from blood and in cfDNA isolated from plasma of patient-matched samples. Similarly to their previous study, the authors assessed DNA methylation by MSP and stratified the samples as positive/negative. In total, the authors detected *SOX17* promoter methylation in 4.3%, 34.5%, and 45.8% of EpCAM-enriched cell fractions, and in 2.0%, 34%, and 40.7% of cfDNA samples, respectively, from healthy donors, patients with operable tumors, and patients with metastatic tumors. In the group of operable breast cancers, the authors reported a significant (*p* = 0.008) correlation between methylation detected in EpCAM-enriched cells and matched cfDNA.

In a third study [119], the same group investigated the association between *BRMS1* promoter methylation and the expression of its encoded protein in CTCs. For that, the authors analyzed the expression of BRMS1 protein and Cytokeratins 8, 18, and 19 by immunofluorescent staining on cytospin preparations of peripheral mononuclear cells (PBMNCs) enriched via Ficoll density gradient centrifugation from peripheral blood of patients with operable breast cancer. The intensity of the BRMS1 staining in CK-expressing cells was semi-quantitatively measured (negative/low/high), using the epithelial breast cancer MCF7 cell line and normal PBMCs as standards. To evaluate *BRMS1* promoter methylation in the CK-positive cells, the authors scratched these from the cytospins, pooled the material, and extracted the DNA for bisulfite treatment and qualitative MSP. They observed a weak correlation between *BRMS1* promoter methylation and lack of protein expression. CTCs lacking BRMS1 protein were detected in eight samples, but methylation of the *BRMS1* promoter could be detected only in four of these. Instead, *BRMS1* gene promoter methylation was detected in one sample without CTCs. A further interesting observation was a high discordance between the methylation statuses of *BRMS1* in CK-positive cells and matched primary tumor samples.

Collectively, these pioneering studies demonstrate that tumor-associated methylation can indeed be detected in cell-fractions enriched for CTCs and provide a first glimpse into the dynamics of epigenetics during human tumor cell dissemination. However, limitations of these three studies were the use of unpurified CTC samples and of the MSP method, which does not provide a quantitative measure of DNA methylation or information on methylation patterns.

A different approach to explore epigenetic features in CTCs was taken by Friedlander and colleagues [134]. These authors enriched viable CTCs by their invasive phenotype from a patient with metastatic castration-resistant prostate cancer (mCRPC) before chemotherapy by taking advantage of the ability of tumor cells to invade a cell-adhesion matrix. This system for capturing CTCs (Vita-Assay™) had been previously described to allow efficient recovery of tumor cells that correlated with clinico-pathological parameters of patients with early stage breast cancer [137], and ovarian cancer [55]. Cells enriched by this technique can be released from the matrix and propagated in culture, or stained for precise enumeration and subsequent molecular characterization, e.g., by array comparative genomic hybridization [137,138]. The invasive cells enriched by this method from the mCRPC patient were then analyzed using a methylation array covering 27,000 CpG-sites [134]. Across all CpGs, the enriched cells presented an average methylation value comparable to that of primary mCRPC tumors, which was higher than in benign prostatic tissues. Notably, 86% of the 1361 loci reported as hypermethylated in mCRPC tumor samples in a previous study [139], were also methylated in the recovered invasive CTCs. The percentage of overlapping loci between these two studies increased to 95% if less stringent criteria were used to call for methylation in the invasive cells. Pathway analyses indicated that methylation in the invasive tumor cells occurred more frequently in genes associated with apoptosis, angiogenesis, and VEGF signaling. This exploratory genome-wide analysis has provided a first insight into global epigenetics of CTCs suggesting that some of the epigenetic programs associated with tumor progression detectable in primary tumors are maintained in CTCs.

In addition to the studies based on *ex vivo* analyses of patient-derived CTCs, interesting epigenetic CTC data were obtained from a syngeneic murine hepatocellular carcinoma (HCC) model. Ogunwobi *et al.* employed this approach to address the role and mechanisms regulating the expression of hepatocyte growth factor (HGF) in CTCs [135]. After orthotopic or subcutaneous implantation, the murine HCC cell-line BNL 1ME A.7R.1 gave rise to detectable tumors and CTCs. The authors established three cell lines from CTCs isolated from these mice for functional and molecular testing. The established CTC cell lines showed increased tumorigenicity and metastatic potential compared to their mother cell line and displayed a more mesenchymal phenotype (increased expression of vimentin, fibronectin, and collagen I, combined with decreased expression of E-cadherin). Concordantly, the hepatocyte growth factor (HGF) and its receptor, the proto-oncogene c-MET, were more strongly expressed in the CTC lines. Accordingly, as in many other cell types [140], HGF induced EMT features in the BNL 1ME A.7R.1 cell line. To explore the mechanisms regulating HGF and c-MET, the authors performed analyses of DNA methylation at the promoter of both respective genes using bisulfite-conversion and high resolution melting analysis, which allows an estimate of methylation across a given PCR amplicon. Indeed, overexpression of HGF and c-MET in the CTC lines was accompanied by a decrease of methylation at their promoters. For c-MET, albeit not for HGF, the results from the high resolution melting analysis were confirmed by the more reliable and quantitative pyrosequencing technique. Although this study was based on an established murine cell line, the *in vivo* results suggest that the passage through the bloodstream may modulate methylation of selected genes in CTCs.

## 5. Outlook and Perspectives

**Figure 2:**
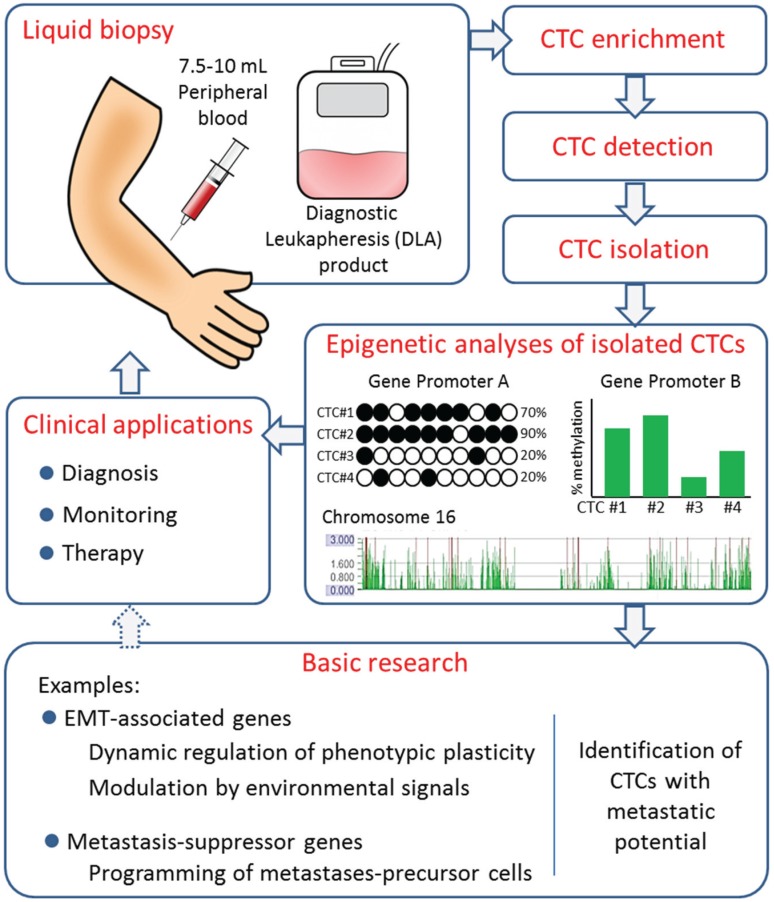
Potential clinical and research applications of epigenetic analyses of CTCs. Standard blood samples or diagnostic leukapheresis products obtained from patients with cancer can be used for CTC enrichment, detection and isolation by different workflows. Isolated CTCs may be analyzed for DNA methylation by different technologies. The information collected from these analyses can be used to address basic molecular mechanisms of metastasis and, in the future, may also prove to be of value for clinical applications.

The analysis of CTCs may provide relevant clinical and biological information on systemic cancer. Whereas phenotypic and genetic profiling have thus far constituted the main objectives of molecular CTC analysis, their epigenetic characterization has been a largely unexplored field. However, the existing data strongly encourages deeper epigenetic analyses of CTCs to address molecular mechanisms of metastasis, and to eventually improve diagnostic, monitor, and therapeutic tools as illustrated in Figure 2.

Considering the high potential of DNA methylation as a biomarker for the diagnosis, prognosis and monitoring of tumors, the low number of studies focusing on methylation analysis of CTCs is surprising. To date, no study of DNA methylation on pure isolated CTCs has been published. The most likely explanation for this state lies in the technical limitations associated with studying DNA methylation on few or single cells, which is necessary to investigate isolated CTCs. Hence, developing more robust protocols for systematic interrogation of DNA methylation on low numbers of cells should be a priority for the field.

Survival and fate of CTCs seem to be closely connected with phenotypical plasticity and the balance between epithelial- and mesenchymal-like states during EMT and MET [69]. Interestingly, existing data largely support the idea that remodeling of DNA methylation is required for EMT and has an impact on various steps of the metastatic cascade [141]. Therefore, DNA methylation is likely to play a key role in CTC survival and regulation of their metastatic potential. In this respect, it seems particularly promising to explore the dynamics of DNA methylation in CTCs and to elucidate to what extent it modulates their phenotypic plasticity. Another interesting aspect that could be studied on CTCs is the DNA methylation response to the different environmental cues [142,143] to which cancer cells are exposed during their journey to distant sites. In particular, it will be important to understand to what extent the blood environment (e.g., plasma composition, interaction with hematogenous cells) may modulate the epigenetic configuration of CTCs and thereby influence their fate. Not least, therapies, especially those by epigenetic drugs like azacytidine, will likely affect the DNA methylation patterns of CTCs. The potential use of CTCs to monitor the efficacy of such therapies should be explored.

In addition to DNA methylation, other epigenetic mechanisms cooperate in global and local regulation of gene transcription (e.g., histone modifications and chromatin accessibility). The study of these epigenetic layers in CTCs will certainly provide additional relevant information on the mechanisms of metastasis, but will require further technology development.

Considering the already reported phenotypic and genetic heterogeneity of CTCs, it appears likely that CTCs constitute an epigenetically heterogeneous population of cells. In this respect, it will be of great importance to explore whether any epigenetic signature can define sub-populations of CTCs with special clinical relevance, allowing, for example, to identify patients that can benefit from specific therapies. In fact, having overcome the initial technical challenge of detecting rare CTCs, the CTC field is now faced with the challenge of dissecting the heterogeneity of CTCs and identifying those among them with the highest clinical and diagnostic utility. Epigenetics could provide a substantial contribution to that aim. However, the proper characterization of their epigenetic status will require a systematic analysis of multiple CTCs at the single-cell level. So far, none of the methods available for analysis of DNA methylation of single cells has been used to analyze isolated CTCs and it is therefore difficult to estimate the performance of these methods in single CTCs. The results obtained from studies in other systems, especially on embryonic development, raise the promise that the technical barriers might soon be overcome and that it will be possible to comprehensively interrogate the epigenome of CTCs in the near future.
